# QTL mapping of volatile compound production in *Saccharomyces cerevisiae* during alcoholic fermentation

**DOI:** 10.1186/s12864-018-4562-8

**Published:** 2018-03-01

**Authors:** Matthias Eder, Isabelle Sanchez, Claire Brice, Carole Camarasa, Jean-Luc Legras, Sylvie Dequin

**Affiliations:** 1 SPO, INRA, SupAgro, Université de Montpellier, F-34060 Montpellier, France; 2 MISTEA, INRA, SupAgro, F-34060 Montpellier, France

**Keywords:** Yeast, Aroma compounds, Metabolites, QTL mapping, Fermentation

## Abstract

**Background:**

The volatile metabolites produced by *Saccharomyces cerevisiae* during alcoholic fermentation, which are mainly esters, higher alcohols and organic acids, play a vital role in the quality and perception of fermented beverages, such as wine. Although the metabolic pathways and genes behind yeast fermentative aroma formation are well described, little is known about the genetic mechanisms underlying variations between strains in the production of these aroma compounds.

To increase our knowledge about the links between genetic variation and volatile production, we performed quantitative trait locus (QTL) mapping using 130 F2-meiotic segregants from two *S. cerevisiae* wine strains. The segregants were individually genotyped by next-generation sequencing and separately phenotyped during wine fermentation.

**Results:**

Using different QTL mapping strategies, we were able to identify 65 QTLs in the genome, including 55 that influence the formation of 30 volatile secondary metabolites, 14 with an effect on sugar consumption and central carbon metabolite production, and 7 influencing fermentation parameters. For ethyl lactate, ethyl octanoate and propanol formation, we discovered 2 interacting QTLs each. Within 9 of the detected regions, we validated the contribution of 13 genes in the observed phenotypic variation by reciprocal hemizygosity analysis. These genes are involved in nitrogen uptake and metabolism (*AGP1, ALP1, ILV6, LEU9*), central carbon metabolism (*HXT3, MAE1*), fatty acid synthesis (*FAS1*) and regulation (*AGP2, IXR1, NRG1, RGS2, RGT1, SIR2*) and explain variations in the production of characteristic sensorial esters (e.g., 2-phenylethyl acetate, 2-metyhlpropyl acetate and ethyl hexanoate), higher alcohols and fatty acids.

**Conclusions:**

The detection of QTLs and their interactions emphasizes the complexity of yeast fermentative aroma formation. The validation of underlying allelic variants increases knowledge about genetic variation impacting metabolic pathways that lead to the synthesis of sensorial important compounds. As a result, this work lays the foundation for tailoring *S. cerevisiae* strains with optimized volatile metabolite production for fermented beverages and other biotechnological applications.

**Electronic supplementary material:**

The online version of this article (10.1186/s12864-018-4562-8) contains supplementary material, which is available to authorized users.

## Background

The aroma of fermented beverages is the result of a complex blend of volatile compounds. In wine, these volatiles originate either directly from grape must or are produced de novo by yeast during alcoholic fermentation. Yeast utilizes the nutrients contained in grape must, which are mainly hexoses, nitrogen and lipid sources, for proliferation, whereas ethanol, CO_2_ and various minor metabolites are produced as byproducts. Many of these metabolites are volatile with sensorial properties, which give wine its vinous character [1]. Although the flavor and aroma profile of wine is influenced by vine environment and management techniques, the choice of yeast strain plays a central role [2].

Higher alcohols and esters are the most abundant groups of fermentative aromas that are produced de novo in yeast metabolism [3]. Higher alcohols can impart a strong, pungent smell and taste when present in higher concentrations, but they result in a fruity character at low concentrations [4]. The formation of higher alcohols is carried out through decarboxylation and reduction of α-keto acids, which derive either from central carbon metabolism or from the transamination of amino acids. Therefore, the synthesis of higher alcohols is linked to both carbon and nitrogen metabolism.

Acetate esters, which are produced by yeast from higher alcohols during fermentation, increase aroma complexity by imparting aromatic notes of pear, apple and banana to general fruitiness [5, 6]. They are synthesized through acetyl transfer from acetyl-CoA to an alcohol by the acetyltransferases Atf1 and Atf2 [7]. Ethyl esters also contribute to global fruitiness perception and are synthesized through acyl transfer from an acyl-CoA to ethanol by the esterases Eeb1 and Eht1 [8]. The carboxylic acid molecules for ethyl ester synthesis predominantly originate from the degradation of α-keto acids or fatty acid synthesis in lipid metabolism.

As a consequence, fermentative aroma can be seen as a complex mix of volatile compounds intimately associated with yeast metabolism. The diversity of yeast strains and variability in the regulation of yeast metabolism have a large impact on their production [9]. Even though biochemical pathways have been established for most of these compounds and major genetic determinants have been identified, the genetic basis for the variation of volatile compound production between strains remains largely unknown.

The formation of several compounds important for wine aroma has been shown to be a quantitative and complex trait, as it is influenced by the contribution of multiple genes [10]. Quantitative trait locus (QTL) mapping has been demonstrated to be a powerful approach for deciphering the genetic bases of numerous complex traits [11, 12] and has been applied in several biotechnological applications. From its first use in enological studies to characterize allelic variants influencing acetic acid production [13], it has been extended to decipher the genomic bases of fermentation parameters [14], the production of main metabolites and residual sugar concentrations [15], nitrogen utilization [16], sulfite production [17] and secondary fermentation [18]. QTL mapping was also used for the detection of genomic regions influencing the production of volatile compounds by yeast during wine alcoholic fermentation [10] using a population of 30 F1-segregants originating from a cross between an *S. cerevisiae* wine and a lab strain. One major QTL and seven minor genomic regions were found to influence the production of different volatile compounds despite high trait heritability. This result suggested that more analytical power is required in order to decipher the genetic bases of the production of volatiles during alcoholic fermentation. The sensitivity of QTL analysis and the ability to find loci with small contributions to phenotype variations can be increased by assessing a larger number of individuals [19]. Moreover, the resolution of the mapping can be improved and nearby QTLs can be unlinked by increasing the recombination rate of the segregants [20]. When multiple loci influence one trait, their contribution to trait variation can either be additive or interacting. Recent studies with a large yeast cross estimated that more than 40% of trait variations in a set of 20 traits could be explained by additive genetic effects, whereas pair-wise genetic interactions contributed to almost 10% of the phenotypic variance [19]. Multiple QTL mapping can not only detect linked QTLs but also provides more statistical power to find unlinked QTLs [21].

In this study, we addressed the complexity of the genetic basis underlying volatile metabolite production using a population of 130 F2-segregants obtained from a cross of two wine strains with different requirements for nitrogen [22]. In addition to performing a genome search for single QTLs, the large segregant population enabled us to increase the analytical strength by performing a search for multiple QTLs. As far as we know, this study is the first analysis of the interaction between loci influencing fermentative aroma formation. We identified a total of 65 QTLs in the genome that influence fermentation parameters and the production of metabolites, including 55 QTLs influencing the formation of 30 volatile secondary metabolites. For the production of ethyl lactate, ethyl octanoate and propanol, we could detect interacting QTLs. Finally, we experimentally validated the role of 13 genes in 9 of the identified genomic regions. These findings provide new information about the production of metabolites of interest due to their sensorial properties or other biotechnological value, such as medium chain fatty acids, fusel acids, higher alcohols and their esters. This opens new perspectives for engineering *S. cerevisiae* strains for broad biotechnological applications.

## Results and discussion

### Phenotyping of strains

**Fig. 1:**
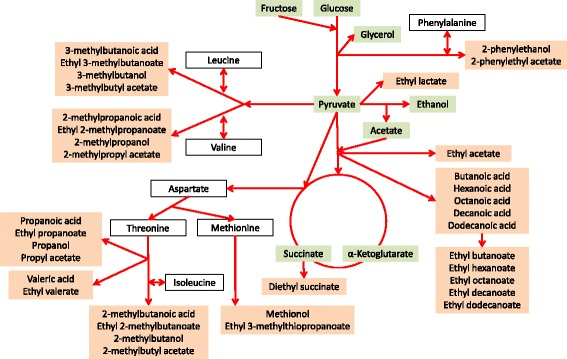
Simplified synthesis pathways of determined metabolites. Main and secondary metabolites determined in this study by HPLC (green) and GC/MS (red)

Using small scale fermenters, the F2-segregant population and both parental strains were phenotyped (Additional files 1 and 2) for the production of 43 extracellular metabolites that originate from nitrogen and central carbon metabolism (Fig. 1). Most traits are normally distributed among the population, indicating that they are under polygenic control (Additional file 2). One exception is the ratio of glucose to fructose after 80% of the fermentation (G/F ratio), which shows a biphasic distribution, revealing the major influence of one locus for this trait. The phenotypes of the parental strains are located within the population of segregants, indicating transgression for most traits, which can be explained by the presence of alleles with opposite impacts on these traits in the parental genomes. Although the differences between the parental strains in their need for nitrogen during fermentation were initially seen as indication of different fermentative aroma formation, a clear dependency of the production of higher alcohols, fusel acids and their esters on the parental nitrogen requirements could not be seen (Additional file 1).

**Fig. 2:**
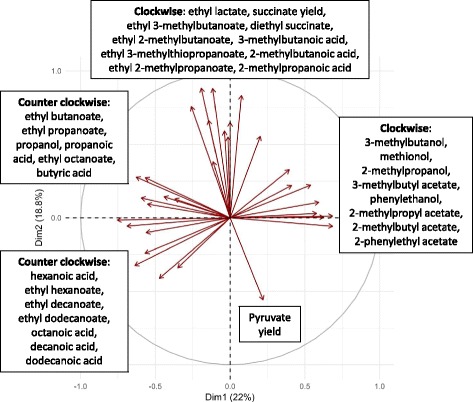
Principle component analysis. PCA of the formation of extracellular metabolites by *S. cerevisiae*. Traits that are less than 2% explained by the first two dimensions of the PCA were excluded (2-methylbutanol, acetate yield, alpha-ketoglutarate yield, ethanol yield, ethyl acetate, glycerol yield, propyl acetate, and valeric acid)

Heritability of the traits was calculated according to Brem et al. (2002) [23] (Additional file 1). With a median of 70.09 and a maximum of 94.35, the determined heritability is high, indicating reproducible phenotyping and a strong genomic influence on trait variations. For the formation of 3-methylbutanol, decanoic acid, diethyl succinate, dodecanoic acid and ethyl dodecanoate, the heritability estimate is almost zero or negative, which might be associated with insufficient analytical reproducibility. We performed a principal component analysis (PCA) to reduce the complexity of the data set for the determined secondary metabolites and to estimate the potential common regulations (Fig. 2). The first two dimensions of the PCA together explain 40.8% of global trait variance. It can be seen that several evaluated compounds are grouped according to their chemical family. The production of all higher alcohols (except propanol and 2-methylbutanol) is correlated, together with the formation of their corresponding acetate esters. The synthesis of these molecules shares a common reaction step, i.e., the decarboxylation and reduction of α-keto acids. Another linked group of volatiles is medium chain fatty acids with their ethyl esters. These compounds share a common pathway, namely, fatty acid synthesis. Interestingly, the formation of fusel acids is not correlated to the production of higher alcohols, although both compounds are metabolized from α-keto acids. This suggests that the reduction or oxidation reactions, which lead to the formation of these compounds from fusel aldehydes, have a strong impact. The pyruvate yield is strongly negatively correlated to the production of ethyl lactate and loosely negatively correlated to the succinate yield and the formation of diethyl succinate. Pyruvate is a metabolic intermediate of both ethyl lactate and succinate formation (Fig. 1).

### Genome-wide identification of QTLs influencing fermentation parameters, main and secondary metabolite production

#### Simple QTL scan

The data obtained from phenotyping and the constructed marker map were used to perform a linkage analysis on 43 quantitative traits, including fermentation parameters, the production of main metabolites and the formation of volatile secondary metabolites. We were able to find a total of 32 QTLs influencing 32 traits (Table 1). The determined logarithm of odds (LOD) scores ranged from 3.41 to 10.49 with a median of 4.35. The highest LOD score of 10.49 was found for the influence of chr4@386.5 on the G/F ratio, which means that 32% of trait variation are explained by the QTL. The strong influence of this locus therefore accounts for the biphasic distribution of the trait. The second highest LOD score of 8.44 was found for a QTL influencing the production of a volatile compound, namely, chr11@127.6 affecting the formation of 2-methylpropanol. Six major QTL regions were detected with LOD scores greater than six for at least one trait, which corresponds to an explained trait variation of more than 20% by these loci.

**Table 1:** QTLs detected with single QTL mapping. List of QTLs with an influence on fermentation parameters, the production of extracellular metabolites and volatile secondary metabolites that were detected with single QTL mapping

	Trait	QTL name	Chromosome	Start position [bp]	End position [bp]	LOD Score
*Fermentation parameters*						
^8^	CO_2_ production rate at t_80%_	chr4@385.5	IV	1,134,839	1,173,812	4.77
^9^	CO_2_ production rate at t_80%_	chr4@410.0	IV	1,198,692	1,246,959	3.69
	CO_2_ production rate at t_80%_	chr10@241.5	X	717,987	726,938	4.67
^20^	Fermentation time t_80%_	chr13@7.9	XIII	20,503	25,723	4.86
^20^	CO_2_ production rate at t_80%_	chr13@7.9	XIII	20,503	25,723	3.81
*Extracellular metabolites after 80% of the fermentation*						
^2^	Pyruvate yield	chr2@172.5	II	507,274	527,387	3.54
^8^	G/F ratio	chr4@386.5	IV	1,153,678	1,173,812	10.49
^9^	G/F ratio	chr4@412.2	IV	1,205,742	1,243,242	5.49
^10^	Pyruvate yield	chr7@20.4	VII	56,448	74,414	6.15
^11^	Pyruvate yield	chr7@156.9	VII	463,981	503,880	3.75
^14^	Pyruvate yield	chr9@58.7	IX	173,782	179,168	4.00
^20^	Pyruvate yield	chr13@7.9	XIII	20,503	25,723	4.80
	Glycerol yield	chr13@19.3	XIII	52,743	75,040	4.82
*Volatile secondary metabolites after 80% of the fermentation*						
^2^	Ethyl butanoate	chr2@166.4	II	488,757	506,771	3.96
	2-methylpropanoic acid	chr3@26.1	III	62,518	111,639	3.96
	Ethyl 2-methylbutanoate	chr4@71.3	IV	211,091	234,153	4.32
^3^	Ethyl 2-methylpropanoate	chr4@82.9	IV	216,058	273,735	3.96
^4^	Methionol	chr4@124.6	IV	365,865	380,035	4.37
^4^	3-methylbutyl acetate	chr4@133.6	IV	376,106	407,166	4.37
^4^	2-methylbutyl acetate	chr4@133.6	IV	397,927	407,166	5.86
^5^	2-phenylethyl acetate	chr4@161.9	IV	455,335	505,548	6.17
^5^	3-methylbutyl acetate	chr4@161.9	IV	455,335	505,548	4.57
^5^	2-methylbutyl acetate	chr4@161.9	IV	478,242	505,548	6.75
^6^	Ethyl lactate	chr4@175.0	IV	521,776	527,398	3.41
^6^	Propanoic acid	chr4@177.5	IV	524,924	545,742	4.00
^6^	Propanol	chr4@177.5	IV	527,398	539,089	5.19
^6^	Propyl acetate	chr4@179.4	IV	527,398	560,742	3.61
^10^	Diethyl succinate	chr7@15.0	VII	40,689	56,448	5.44
^10^	Ethyl 3-methylthiopropanoate	chr7@25.5	VII	50,239	87,729	4.47
^11^	Ethyl lactate	chr7@161.6	VII	458,995	518,880	3.97
	Dodecanoic acid	chr7@175.5	VII	494,396	548,880	4.76
^12^	Dodecanoic acid	chr7@195.5	VII	578,880	601,380	3.68
	2-phenylethyl acetate	chr7@294.6	VII	853,536	885,989	4.14
^17^	Octanoic acid	chr11@29.5	XI	77,969	117,578	3.99
^17^	Hexanoic acid	chr11@29.5	XI	82,548	115,238	6.94
^17^	Ethyl hexanoate	chr11@35.5	XI	97,410	115,238	5.43
^17^	Ethyl dodecanoate	chr11@41.8	XI	115,238	145,211	4.29
^17^	2-phenylethyl acetate	chr11@42.5	XI	125,453	132,044	4.29
^18^	2-methylpropyl acetate	chr11@123.8	XI	366,406	391,690	6.63
^18^	2-methylpropanol	chr11@127.6	XI	371,345	400,712	8.44
^18^	3-methylbutanol	chr11@132.7	XI	380,437	403,181	3.56
^18^	2-methylpropanoic acid	chr11@134.4	XI	400,712	405,331	4.64
	2-methylpropanol	chr11@158.4	XI	470,852	477,578	3.80
^19^	Propyl acetate	chr12@222.6	XII	662,035	699,182	4.83
^19^	Propanol	chr12@226.9	XII	662,035	691,268	4.08
	Valeric acid	chr13@102.0	XIII	296,288	312,983	5.87
^21^	Propanoic acid	chr14@41.9	XIV	119,900	146,614	4.67
^21^	Propanol	chr14@43.9	XIV	119,900	146,614	7.03
^21^	Propyl acetate	chr14@46.9	XIV	124,114	150,370	6.19
^21^	Valeric acid	chr14@48.9	XIV	125,823	150,370	7.72
	Propanoic acid	chr14@58.9	XIV	160,420	185,626	3.83
	Valeric acid	chr14@81.8	XIV	233,520	259,114	3.88
	Ethyl 3-methylbutanoate	chr15@77.3	XV	212,898	239,482	3.76
^22^	Ethyl decanoate	chr15@139.0	XV	409,364	431,700	3.97
^22^	Ethyl octanoate	chr15@142.3	XV	414,810	438,628	5.02
^23^	3-methylbutanoic acid	chr15@162.7	XV	485,607	511,993	4.29
^23^	2-phenylethyl acetate	chr15@176.5	XV	511,993	545,871	3.56
	Diethyl succinate	chr15@297.3	XV	879,033	901,450	4.46
	3-methylbutanoic acid	chr16@191.9	XVI	552,371	593,439	3.83
^25^	Diethyl succinate	chr16@303.9	XVI	899,570	920,003	4.08
^25^	Ethyl 3-methylbutanoate	chr16@303.9	XVI	904,961	917,224	3.81
^25^	Ethyl 2-methylbutanoate	chr16@304.1	XVI	904,961	917,224	3.79

Single traits that are influenced by the same QTL (under the condition that the distance between detected peaks is less than 10 cM; in combination with Table 2) are indicated with superscript numbers in the left column of the table

Globally, these QTLs were distributed over the whole genome, with exception of chromosomes I, V, VI and VIII. The size of the identified regions ranged from 5.2 kb to 65.7 kb with a median of 33.2 kb. The detected regions contained between 4 and 28 genes. Four QTLs were detected for both evaluated traits of the fermentation kinetics, the fermentation time (t_80%_) and CO_2_ production rate at t_80%_. Eight QTLs were found for the concentration of extracellular main metabolites at t_80%_. These QTLs influenced three traits, which were glycerol yield, pyruvate yield and the G/F ratio. The most QTLs were detected for the formation of volatile secondary metabolites, namely, 28 QTLs influencing the formation of 27 volatiles. This included the production of characteristic sensorial compounds, such as 2-methylbutyl acetate, 3-methylbutanol and 3-methylbutyl acetate, and industrially relevant chemicals, such as higher alcohols and organic acids.

The detected QTLs were compared with loci found in QTL mapping studies of similar traits [10, 13–15, 24]. Only QTL chr7@161.6, which influences ethyl lactate formation, co-localizes with *PMA1*, a plasma membrane P2-type H + -ATPase that was shown by Abt et al. (2016) to be the responsible gene in a QTL affecting ethyl acetate production. More QTLs were in common with the findings of Rossouw et al. (2008), who used a comparative approach of combining transcriptomics and exo-metabolome analysis to predict candidate genes with a role in aroma profile modification [25]. Several of the genes proposed by Rossouw et al. (2008), e.g., *ALP1, ILV6, LEU1, LEU2* and *LEU9*, were included in QTLs that we detected for the same or closely related traits. As the parent strains were selected because of their different need for nitrogen during fermentation, we compared the detected QTLs with genomic regions found by Brice et al. (2014) to influence nitrogen requirement using the same parental strains [16]. No match could be seen for QTLs influencing fermentative aroma formation and nitrogen requirement, which could possibly be explained by the differences in fermentation conditions.

#### Double and multiple QTL scan

To search for additional minor QTLs and to assess genetic interactions between our detected regions, we performed a two-dimensional, two-QTL scan and a multiple QTL search for all traits. The analyses confirmed 24 QTLs that were already found with the single QTL mapping and proposed 36 additional loci (Table 2). With the double QTL mapping, we found significant evidence for an interacting QTL pair at positions chr10@88.5 and chr11@51.7, influencing the formation of ethyl octanoate and accounting for 10.29% of trait variation. Another interacting QTL pair, chr2@181.0 and chr16@304.0, was found to influence the production of ethyl lactate, explaining 10.17% of trait variation. Multiple QTL mapping proposed interacting regions for a wide range of traits. However, their contributions to the respective phenotypes were low, with LOD scores of generally less than two. Due to penalization of the LOD score for more complex models of interaction, solely additive models were found to be more significant for all traits. This indicates that our number of segregants was still insufficient to achieve the statistical power required for the determination of QTL interactions. However, for the production of propanol the three involved QTLs, chr4@176.9, chr12@233.1 and chr14@45.8, could be detected with all three mapping strategies, giving strong evidence for their validity. Although models consisting of viewer QTLs were seen to be more probable by the multiple QTL mapping, the most likely model containing all three loci was an additive model with an interaction between chr4@176.9 and chr12@233.1. This indicates a remaining probability for the proposed interaction, which was calculated to potentially account for 5.58% of trait variation.

**Table 2:** QTLs detected with double and multiple QTL mapping. QTLs with an influence on fermentation parameters and the production of extracellular metabolites and volatile secondary metabolites that were additionally detected with double and multiple QTL searches

	Trait	QTL name	Chromosome	Peak [bp]	LOD Score
	Diethyl succinate	chr1@64.7	I	194,100	4.23
^1^	Ethyl 2-methylbutanoate	chr2@116.2	II	348,600	4.13
^1^	Hexanoic acid	chr2@122.1	II	366,300	2.54
^2^	Ethyl lactate	chr2@181.0	II	543,000	4.78
	Ethyl 2-methylpropanoate	chr3@78.8	III	236,400	2.84
^3^	Pyruvate yield	chr4@91.9	IV	275,730	2.39
^3^	Ethyl butanoate	chr4@92.0	IV	275,985	3.63
^4^	Ethyl hexanoate	chr4@132.0	IV	396,000	2.59
^5^	2-methylpropyl acetate	chr4@165.8	IV	497,400	2.42
^6^	Diethyl succinate	chr4@175.0	IV	525,000	2.99
^6^	Ethyl octanoate	chr4@175.0	IV	525,000	2.14
	Dodecanoic acid	chr4@298.0	IV	894,000	4.60
	Valeric acid	chr4@324.9	IV	974,700	2.50
^7^	2-methylpropanol	chr4@348.9	IV	1,046,700	3.36
^7^	3-methylbutanol	chr4@348.9	IV	1,046,730	3.66
^9^	Pyruvate yield	chr4@411.9	IV	1,235,730	2.54
	G/F ratio	chr6@62.0	VI	186,000	3.90
	Ethyl 3-methylbutanoate	chr6@85.2	VI	255,600	4.14
^12^	Decanoic acid	chr7@198.0	VII	594,000	3.65
^13^	2-methylpropyl acetate	chr9@16.9	IX	50,700	2.09
^13^	Dodecanoic acid	chr9@17.6	IX	52,800	3.20
	2-methylbutyl acetate	chr9@32.6	IX	97,800	1.75
^14^	Dry weight	chr9@60.0	IX	180,066	3.67
^14^	Ethyl propanoate	chr9@67.5	IX	202,500	2.88
^14^	Propanol	chr9@67.5	IX	202,500	1.85
	2-phenylethyl acetate	chr9@101.1	IX	303,300	2.25
	Propyl acetate	chr10@27.2	X	81,600	1.53
^15^	Ethyl hexanoate	chr10@61.3	X	183,900	2.97
^15^	Hexanoic acid	chr10@61.3	X	183,900	2.78
	Ethyl octanoate	chr10@88.5	X	265,500	5.24
	Propanol	chr10@104.7	X	314,100	2.08
^16^	2-methylpropanol	chr10@213.1	X	639,300	2.29
^16^	2-methylpropyl acetate	chr10@221.7	X	665,100	4.04
^16^	2-methylbutyl acetate	chr10@228.1	X	684,300	2.34
^16^	3-methylbutyl acetate	chr10@228.8	X	686,400	2.97
^17^	Decanoic acid	chr11@29.2	XI	87,600	3.95
^17^	Ethyl decanoate	chr11@34.2	XI	102,438	2.65
^17^	t_80%_	chr11@43.2	XI	129,600	3.10
^17^	Ethyl octanoate	chr11@51.7	XI	155,100	4.95
	Glycerol yield	chr11@70.0	XI	210,000	2.72
	G/F ratio	chr11@85.6	XI	256,800	2.65
	Ethyl propanoate	chr11@107.7	XI	323,100	3.78
^18^	3-methylbutanoic acid	chr11@134.4	XI	403,170	3.68
	2-methylpropyl acetate	chr12@98.3	XII	294,900	3.15
	Ethyl 2-methylbutanoate	chr12@123.8	XII	371,400	3.34
	Ethyl propanoate	chr12@139.6	XII	418,800	3.67
	CO_2_ production rate at t_80%_	chr12@257.3	XII	771,900	2.47
	2-methylpropanol	chr12@317.1	XII	951,300	3.08
	G/F ratio	chr13@164.5	XIII	493,500	4.99
	Propyl acetate	chr13@248.3	XIII	744,900	2.11
	2-phenylethyl acetate	chr13@304.0	XIII	912,000	2.55
^21^	Dodecanoic acid	chr14@36.3	XIV	108,900	4.69
	Ethyl 3-methylthiopropanoate	chr14@227.0	XIV	681,000	3.42
^22^	2-phenylethanol	chr15@132.0	XV	396,000	1.62
^23^	2-methylbutyl acetate	chr15@172.0	XV	516,000	2.55
	Ethyl hexanoate	chr15@265.4	XV	796,200	4.47
	Ethyl 3-methylbutanoate	chr15@304.4	XV	913,200	2.77
	2-methylpropanoic acid	chr15@314.4	XV	943,200	2.95
	2-phenylethyl acetate	chr15@352.0	XV	1,056,000	2.85
^24^	2-methylpropanol	chr16@13.8	XVI	41,400	4.06
^24^	2-methylbutyl acetate	chr16@16.4	XVI	49,200	2.78
^24^	G/F ratio	chr16@18.3	XVI	54,900	2.75
	Valeric acid	chr16@116.5	XVI	349,500	2.04
^25^	Ethyl lactate	chr16@304.0	XVI	912,000	7.00

Single traits that are influenced by the same QTL (under the condition that the distance between detected peaks is less than 10 cM; in combination with Table 1) are indicated with superscript numbers in the left column of the table

Combining the results from the single and multiple QTL mapping, each trait is influenced by a median of 3 QTLs, ranging from 1 to 7. The best explained trait is the G/F ratio with 6 detected loci accounting for 61.0% of determined trait variation. Regarding volatile formation, the difference in 2-methylpropanol production can be best elucidated, with 6 identified QTLs explaining 54.5% of trait variation. Several QTLs display pleiotropic effects, as they influence many traits and can therefore be considered “hotspots”. The region affecting the most traits is chrXI:77,969..155,100, which influences the fermentation time (t_80%_) and the formation of eight volatile compounds, namely, hexanoic acid, octanoic acid, decanoic acid, and their corresponding ethyl esters as well as ethyl dodecanoate and 2-phenylethyl acetate. Twenty-four other QTLs were found to influence more than one trait. This is often the case for the production of related compounds and indicates common regulation, which was already concluded by PCA. Examples for jointly influenced traits are sugar consumption and the CO_2_ production rate, pyruvate yield and the formation of ethyl esters, the production of 2-methylpropanol and 3-methylbutanol, and the formation of several acetate esters.

### Validation of genomic regions involved in metabolic traits

For the validation of single QTLs and the identification of impacting allelic variants within the corresponding regions, 19 genes in 10 QTLs were further evaluated using RHA (Table 3). These target genes were chosen since they contained non-synonymous SNPs between the parent cells and were suspected to play a role in the detected traits, as their biologic functions were mostly connected to central carbon metabolism or nitrogen uptake and metabolism. In 9 QTLs, we could identify 13 genes that influence hexose transport, CO_2_ production rate and the formation of medium chain fatty acids, fusel acids, higher alcohols, and their corresponding esters (Table 3).

**Table 3:** Validated allelic variants in detected QTLs. Selected target genes for the validation of QTLs influencing fermentation kinetics, substrate consumption and the production of fermentative aromas; differences caused by the allelic gene variants regarding the influenced traits were detected by RHA and are given as the ratio of phenotype MTF2621 to phenotype MTF2622

QTL name	Trait	Evaluated genes	Different impact of allele on trait as MTF2621/MTF2622 [factor]
chr2@166.4	Ethyl butanoate Ethyl lactate Pyruvate yield	*AGP2*	0.79*** ethyl lactate
chr3@26.1	2-methylpropanoic acid	*AGP1*	1.26* 2-methylpropanoic acid
		*ILV6*	no effect
chr4@71.3	Ethyl 2-methylbutanoate Ethyl butanoate Ethyl 2-methylpropanoate Pyruvate yield	*YDL124W*	no effect
chr4@133.6	2-methylbutyl acetate 3-methylbutyl acetate Ethyl hexanoate Methionol	*SIR2*	0.77* 3-methylbutyl acetate 0.78** ethyl hexanoate
chr4@177.5	Ethyl lactate Ethyl octanoate Diethyl succinate Propanoic acid Propanol Propyl acetate	*NRG1*	1.10* propanol
chr4@386.5	CO_2_ production rate at t_80%_	*HXT3*	1.07* CO_2_ production rate
	G/F ratio		1.86** G/F ratio
		*HXT6*	no effect
		*HXT7*	no effect
chr11@29.5	2-phenylethyl acetate Ethyl decanoate Ethyl dodecanoate Ethyl hexanoate Ethyl octanoate Decanoic acid Hexanoic acid Octanoic acid t_80%_	*ACP1*	no effect
		*FAS1*	0.81** ethyl hexanoate 0.82** decanoic acid 0.84**hexanoic acid 0.89** octanoic acid
		*FAT3*	no effect
		*PXA2*	no effect
chr11@127.6	2-methylpropanoic acid 2-methylpropanol 2-methylpropyl acetate 3-methylbutanoic acid 3-methylbutanol	*IXR1*	1.14** 2-methylpropanol 1.16* 2-methylpropanoic acid
		*MAE1*	1.43** 2-methylpropanoic acid 1.67*** 2-methylpropanol 1.27*** 3-methylbutanoic acid 1.40* 3-methylbutanol
		*RGT1*	1.15*** 2-methylpropanol
chr12@226.9	Propanol Propyl acetate	whole region	no effect
chr14@48.9	Dodecanoic acid Propanoic acid Propanol Propyl acetate Valeric acid	*ALP1*	0.90* dodecanoic acid 1.07** propanol 1.26*** valeric acid
chr15@176.5	2-methylbutyl acetate 2-phenylethyl acetate 3-methylbutanoic acid	*LEU9*	1.08* 2-phenylethyl acetate
		*RGS2*	1.21* 2-methylbutyl acetate 0.83* 2-phenylethyl acetate

(*p*-value: ns (not significant) > 0.05, * ≤ 0.05, ** ≤ 0.01, *** ≤ 0.001)

#### Hexose transporter Hxt3 influences sugar utilization

Hexose transport is a limiting step for alcoholic fermentation speed [26]. QTL chr4@386.5, which influences the CO_2_ production rate and G/F ratio, contains three hexose transporter genes, *HXT3*, *HXT6* and *HXT7*. We evaluated these genes individually by RHA. As the sequences of *HXT6* and *HXT7* are nearly identical, we assessed the effect of both genes together. Variation in *HXT3* was found as the sole effect influencing the CO_2_ production rate and the G/F ratio (Additional file 3). The MTF2621 allele of the gene increased the CO_2_ production rate by a factor of 1.07 and increased the G/F ratio by a factor of 1.86. An effect of this allelic variation on the production of determined volatiles could not be detected. A variant of *HXT3* has already been described in the literature by Guillaume et al. (2007) to have a higher affinity for fructose [27] and was detected among flor strains [28]. This variant originated through recombination between the orthologs *HXT1* and *HXT3*. Except for SNP T1411A, which results in amino acid change L471I, the MTF2621 allele of *HXT3* is identical to the variant described by Guillaume et al. (2007). We can therefore suggest that the allelic variants of *HXT3* are the driving factor behind the biphasic distribution of the G/F ratio among the segregants.

#### The formation of medium chain fatty acids and their ethyl esters is influenced by Agp2, Fas1 and Sir2

Ethyl esters of medium chain fatty acids provide floral and fruity notes to fermented beverages. In QTL chr4@133.6 and QTL hotspot chr11@29.5, which influence the formation of medium chain fatty acids and their ethyl esters, we identified *SIR2* and *FAS1* as causative genes (Table 3). In chr2@166.4, a QTL impacting ethyl butanoate production with a lower significance (LOD 3.96), *AGP2* was found to modulate the formation of butanoic acid, the substrate for ethyl butanoate.

**Fig. 3:**
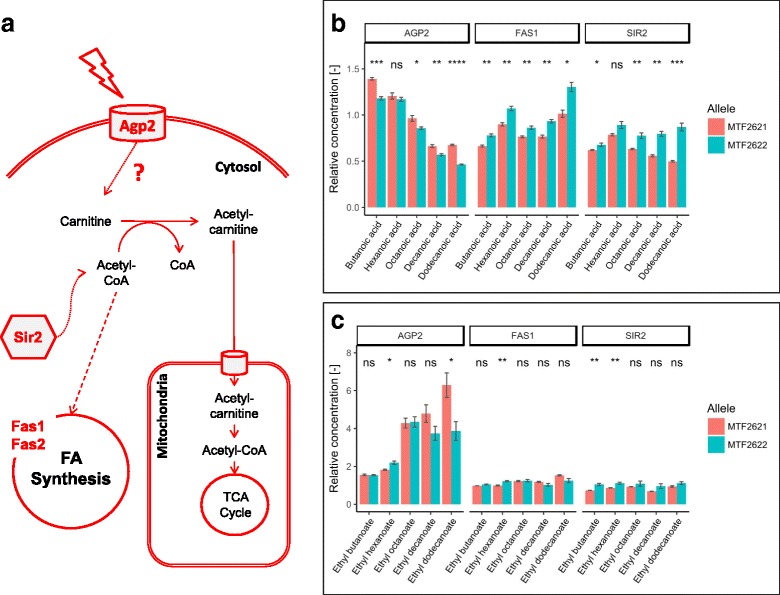
Effect of validated variants on medium chain fatty acid formation. Simplified pathway of fatty acid synthesis by the enzymes Fas1 and Fas2, which is dependent on intracellular acetyl transport (**a**). Allelic effect of the enzymes Agp2, Fas1 and Sir2 on the formation of fatty acids (**b**) and fatty acid ethyl esters (**c**) as determined by RHA. Concentrations are given in relation to the heterozygote of the parental strains MTF2621 and MTF2622. (*p*-value: ns (not significant) > 0.05, * ≤ 0.05, ** ≤ 0.01, *** ≤ 0.001, **** ≤ 0.0001)

Fatty acids are synthesized from the repeated condensation of malonyl-CoA and acetyl-CoA, which is carried out by fatty acid synthetase (FAS). The FAS complex consists of the beta subunit Fas1 and the alpha subunit Fas2 [29]. Fas1, which was found to regulate the expression of *FAS2* [30], possesses four independent enzymatic functions, i.e., acetyl transferase, enol reductase, dehydratase and malonyl/palmitoyl transferase [31]. The parental allelic variants of *FAS1* differ in three non-synonymous SNPs (Table 4), of which one, SNP I1970V, lies in the malonyl-CoA-acyl carrier protein transacylase domain of the protein. The MTF2621 allele of Fas1 causes a significant decrease in the formation of hexanoic acid, ethyl hexanoate, valeric acid, octanoic acid, decanoic acid and dodecanoic acid by a factor of 0.78–0.89 (Fig. 3). Therefore, we can suggest that the MTF2621 allele of *FAS1* is less active than the MTF2622 allele, and thus leads to a decreased synthesis rate of fatty acids.

**Table 4:** Non synonymous SNPs between allelic variants. Differences in the amino acid (AA) sequence of the expressed protein resulting from non-synonymous SNPs between the allelic variants of the evaluated target genes. Comparison of the strains MTF2621 and MTF2622 with the *S. cerevisiae* reference strain S288C

Gene	AA position	S288C	MTF2621	MTF2622
*AGP1*	7	P	P	L
	24	G	E	G
	142	N	S	N
	316	V	V	A
	370	F	L	F
	466	I	L	I
	540	L	I	L
	597	D	N	D
*AGP2*	256	H	Y	H
*ALP1*	126	V	V	A
*FAS1*	1504	V	A	V
	1715	V	A	V
	1970	V	I	V
*ILV6*	4	S	L	S
	56	A	P	A
*IXR1*	45	T	T	A
	65	Q	Q	–
	93	Y	Y	F
	104	–	ATTTTT	–
	291	M	M	L
	570	QQ	QQ	–
*LEU9*	76	D	D	H
	176	S	S	Y
*MAE1*	605	I	I	V
*NRG1*	129	P	H	P
	156	T	S	T
*RGS2*	99	Y	N	Y
*RGT1*	326	L	L	P
	717	I	I	V
	722	P	P	A
	729	S	S	N
*SIR2*	178	Q	Q	H
	201	S	G	S

The gene *SIR2* encodes an NAD + −dependent deacetylase involved in chromatin silencing [32]. The allelic variants of *SIR2* differ in two non-synonymous SNPs (Table 4). The MTF2621 allele of the gene causes a decrease in the formation of hexanol, octanoic acid, decanoic acid, dodecanoic acid, ethyl butanoate and ethyl hexanoate up to a factor of 0.57 (Fig. 3). In addition, the extracellular concentration of acetate was decreased by a factor of 0.8 (Additional file 3). Sir2 was found to influence the expression of the acetyl-CoA synthase *ACS2* [33], and a regulating function by Sir2 on the activity of acetyl-CoA synthase enzymes by deacetylation was proposed by Starai et al. (2003) [34]. Furthermore, Casatta et al. (2013) demonstrated that a null mutant of *SIR2* showed increased acetate metabolism and a lower excretion of acetate to the medium [35]. Based on our observations, we can therefore suggest that the MTF2621 variant of Sir2 has lower deacetylase activity compared to the other variant, which results in decreased expression of *ACS2* and reduced activation of acetyl-CoA synthases. Consequently, this reduced activity leads to a lower availability of acetate and acetyl-CoA for fatty acid synthesis and elongation.

**Fig. 4:**
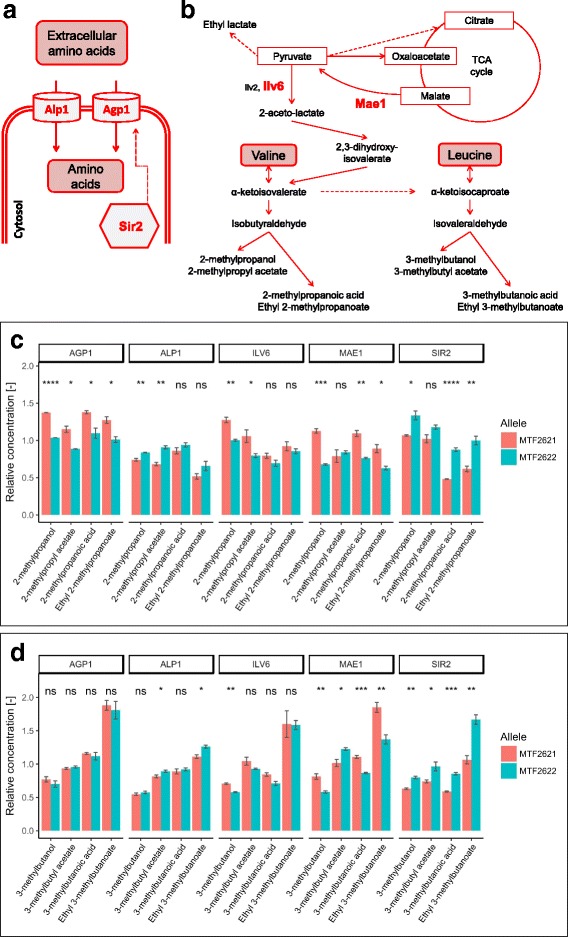
Effect of validated variants on of higher alcohol and fusel acid formation. Amino acids are transported into the cell by Agp1 and Alp1. The expression of *AGP1* is influenced by Sir2 (**a**). Simplified synthesis pathway of fermentative aromas connected to valine and leucine metabolism (**b**). Allelic effect of the involved enzymes Agp1, Alp1, Ilv6, Mae1 and Sir2 on the formation of volatiles deriving from α-ketoisovalerate (**c**) and α-ketoisocaproate (**d**) as determined by RHA. Concentrations are given in relation to the heterozygote of the parental strains MTF2621 and MTF2622. (*p*-value: ns (not significant) > 0.05, * ≤ 0.05, ** ≤ 0.01, *** ≤ 0.001, **** ≤ 0.0001)

Other small but significant influences of *SIR2* can be seen in the formation of 2-methylpropanol, 3-methylbutanol and other degradation products of α-keto acids, with the MTF2621 allele leading to a decrease of these compounds (Fig. 4). Sir2 was found to modulate the expression of the amino acid permease *AGP1* [36]. Altered expression of *AGP1* could impact the nitrogen assimilation and thus the formation of amino acid related fermentative aromas. Furthermore, as Sir2 is dependent on the cofactor NAD+, its altered activity could influence redox homeostasis of the cell. Redox imbalances were reported to significantly affect the production of fermentative aromas by *S. cerevisiae* [37].

*AGP2* encodes a plasma membrane protein that is involved in the uptake of carnitine and polyamines [38, 39]. Carnitine is important for intracellular acetyl transport between cellular compartments [40], and the level of carnitine can therefore affect the availability of acetyl-CoA for fatty acid synthesis. However, carnitine is not present in the synthetic medium used in this study. Agp2 positively regulates the expression of various proteins involved in substrate transport and other biological processes, and might also act as a sensor of environmental signals [41]. One non-synonymous SNP was found to distinguish the two parental variants (Table 4), and it is located in the extracellular region of the protein. The MTF2621 allele of *AGP2* causes an increase in the formation of butanoic acid, decanoic acid, dodecanoic acid and ethyl dodecanoate up to a factor of 1.63 (Fig. 3). We suggest that the reported SNP in *AGP2* causes a higher formation rate of fatty acids for MTF2621, although the causative function remains unclear.

#### The formation of higher alcohols, fusel acids and their esters is influenced by Agp1, Ilv6, Mae1

Higher alcohols, fusel acids and especially their esters are essential fermentative aroma components that provide notes ranging from fruity to flowery. We identified *MAE1* in chr11@127.6, the QTL with the highest LOD score for volatile compounds, which influences the formation of five higher alcohols, fusel acids and acetate esters (Table 3). The enzyme Mae1 catalyzes the oxidative decarboxylation of malate to pyruvate [42]. Pyruvate is a precursor for the synthesis of the amino acids alanine, isoleucine, leucine and valine [43]. An intermediate of valine and leucine biosynthesis, α–ketoisovalerate, can also be degraded to 2-methylpropanol and 2-methylpropanoic acid or to 3-methylbutanol and 3-methylbutanoic acid via α–ketoisocaproate (Fig. 4). The allelic variants of *MAE1* differ in one non-synonymous SNP (Table 4), which is located in the NAD-binding domain of the protein. Furthermore, 5 SNPs in the 1000-bp upstream region of the gene affect predicted binding motifs for the proteins Azf1, Mot3, Rtg1, Rtg3, Stp1 and Stp2 (Additional file 4). The hemizygote carrying only the MTF2621 allele of *MAE1* shows increased formation of 2-methylpropanol, 3-methylbutanol, 2-methylpropanoic acid and 3-methylbutanoic acid by up to a factor of 1.67 (Fig. 4). We can suggest that the MTF2621 allele of *MAE1* is superior to the MTF2622 allele and induces an increased flux of malate to pyruvate, leading to higher formation of α–keto acids and their degradation products. This proposal is further supported by an observed increased formation of ethyl lactate (Additional file 3), which is also derived from pyruvate (Fig. 1).

RHA detected several other minor influences of *MAE1* on traits that were not found by QTL mapping. The MTF2621 allele of the gene leads to a slightly higher production of 2-phenylethanol by a factor of 1.18 (Additional file 3). Mae1 was found to interact with Aro1 [44], an enzyme catalyzing several steps of the chorismate pathway leading to the synthesis of aromatic amino acids, such as phenylalanine [45]. In addition, the MTF2621 allele of Mae1 leads to a decrease of several acetate esters and medium chain fatty acids up to a factor of 0.8 (Fig. 4 and Additional file 3) and to an increase in the extracellular concentration of acetate by a factor of 1.28 (Additional file 3). These effects are consistent with the fact that Mae1 physically interacts with Acc1 [44], an acetyl-CoA carboxylase that is involved in the regulation of acetyl-CoA and in the biosynthesis of medium and long chain fatty acids [46, 47].

The gene *AGP1*, which encodes a low affinity amino acid permease for asparagine and glutamine [48], was validated in QTL chr3@26.1 with an influence on the formation of 2-methylpropanoic acid. The two allelic variants of *AGP1* differ in 8 non-synonymous SNPs (Table 4), of which three lie in cytoplasmic domains of the protein and 5 in transmembrane domains. Another SNP is located in the 1000-bp upstream region of the gene, affecting the predicted binding motif for Ume6 (Additional file 4). The hemizygote carrying the MTF2621 allele of the gene shows a formation of 2-methylpropanol, 2-methylpropyl acetate, 2-methylpropanoic acid and ethyl 2-methylpropanoate increased by a factor of 1.26–1.32 (Fig. 4). Agp1 was found to transport valine to a lower extent [48]. Valine can be degraded to α-ketoisovalerate and then to 2-methylpropanol and 2-methylpropanoic acid by the Ehrlich pathway (Fig. 4). We hypothesize that the reported SNPs lead to higher affinity of the MTF2621 allele of Agp1 for valine, leading to a higher level of this amino acid in the cell. Another possible explanation is a different influence of the alleles on the transport of glutamine, which is important for the transamination of α-keto acids in the cell. In this scenario, a reduced intracellular level of glutamine could lead to a decreased transamination of α-ketoisovalerate, which can therefore be degraded to 2-methylpropanol and 2-methylpropanoic acid. While this would also affect the transamination of other α-keto acids and therefore the production of several higher alcohols or fusel acids, a significant, but small, influence of *AGP1* could only be additionally detected on the production of 2-phenylethanol.

In the same QTL (chr3@26.1), the variants of *ILV6* did not show significant differences in the formation of 2-methylpropanoic acid, but they did in the formation of the related higher alcohol 2-methylpropanol. Ilv6 is a regulatory subunit of the acetolactate synthase Ilv2, which catalyzes the first step of valine and leucine biosynthesis [49]. The allelic variants of *ILV6* differ in two non-synonymous SNPs (Table 4). SNP L4S lies in the N-terminal signal peptide domain of the protein, whereas SNP P56A is within a non-cytoplasmic domain. Another SNP is located in the 1000-bp upstream region and causes a loss of the predicted binding motifs for Msn2, Msn4, Nrg1 and Rph1 in strain MTF2621 (Additional file 4). The MTF2621 allele of *ILV6* leads to an increase in the formation of 2-methylpropanol and 3-methylbutanol by a factor of 1.24 (Fig. 4). Therefore, we can hypothesize that the MTF2621 allele of *ILV6* stimulates a higher synthesis rate of acetolactate, which could result in higher synthesis of α–ketoisovalerate, including its degradation products.

#### The formation of propanol is influenced by Alp1 and Nrg1

We assessed the three partly interacting genomic regions that were detected with the single, double and multiple QTL mapping to affect the production of propanol and related compounds (Table 3). *NRG1* and *ALP1* were validated in QTL chr4@177.5 and QTL chr14@43.9, respectively. As no clear candidate gene was identified in QTL chr12@226.9, we assessed the whole region by RHA; however, no significant impact could be detected for the production of propanol or propyl acetate. The QTL is likely to interact with chr4@177.5, and the negative validation of chr12@226.9 might indicate a possible epistatic interaction. Furthermore, chr12@226.9 is the weakest QTL of the three assessed loci, with an LOD score of 4.08 (Table 1), which could have hindered the validation.

**Fig. 5:**
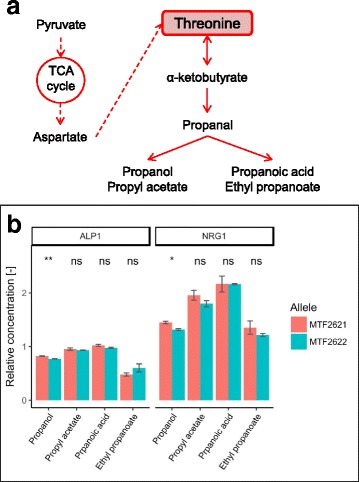
Effect of validated variants on propanol formation. Simplified synthesis pathway of fermentative aromas connected to threonine metabolism (a). Allelic effect of the involved enzymes Alp1 and Nrg1 on the formation of volatiles derived from α-ketobutyrate as determined by RHA (**b**). Concentrations are given in relation to the heterozygote of the parental strains MTF2621 and MTF2622. (*p*-value: ns (not significant) > 0.05, * ≤ 0.05, ** ≤ 0.01)

Propanol and propanoic acid derive from the decarboxylation of α-ketobutyrate and the oxidation or reduction of the resulting propionaldehyde [50]. α-Ketobutyrate is produced from the transamination of threonine, which is taken up from the medium as a nitrogen source or can be metabolized from pyruvate via aspartate through the amino acid pathway (Fig. 5). It was shown, however, that the formation of propanol is mainly limited to the beginning of wine fermentation when nitrogen is present in the must and is dependent on the initial amount of available nitrogen [51].

The protein Nrg1 is a transcriptional regulator of glucose repressed genes [52, 53] and mediates a set of stress responsive genes [54]. The parental allelic variants of *NRG1* differ in two non-synonymous SNPs (Table 4), which are both located in the transcriptional repressor protein “yy” domain. The MTF2621 variant of the gene leads to an increase in propanol production by a factor of 1.10, whereas no significant effect could be detected in the formation of related compounds (Fig. 5). The repressive function of Nrg1 is inhibited by Snf1; therefore, it is suspected to have a role in the response to nitrogen limitation [55]. Furthermore, Nrg1 was found to influence the expression of *BAT1*, a mitochondrial aminotransferase involved in branched amino acid synthesis and Ehrlich pathway catabolism [56]. With regard to this finding, we propose that the allelic variants of Nrg1 show a different response to nitrogen limitation, which affects the expression of *BAT1*, leading to a lower transamination rate of valine, leucine and isoleucine taken up from the medium. In this scenario, the overall availability of nitrogen for metabolism would be influenced, which therefore influences the synthesis of propanol.

The gene *ALP1* encodes a permease for cationic amino acids [57, 58]. The parental variants differ in one non-synonymous SNP (Table 4), which is located in a transmembrane domain. The MTF2621 variant increases the production of propanol by a factor of 1.07 (Fig. 5). We can suggest that this variant of Alp1 leads to an increased uptake of amino acids from the medium at the beginning of the fermentation, which explains higher propanol formation. This hypothesis is supported by a significant decrease in fermentative aromas derived from α-ketoisovalerate and α-ketoisocaproate for the MTF2621 allele of the gene (Fig. 4). The opposite correlation is reported in the literature, in which a lower overall intracellular concentration of nitrogen leads to a higher level of fermentative aroma production due to a lower transamination rate of α-keto acids derived from central carbon metabolism [59, 60].

## Conclusion

In this study, we confirm the potential of QTL analysis for deciphering the impact of genetic variation on the production of volatile metabolites by *Saccharomyces cerevisiae* during alcoholic fermentation. We were able to enlarge the analytical power of the approach compared to previous studies by using a comparatively large number of 130 segregants originating from a cross of two wine strains and by increasing the recombination rate of the segregants. This approach enabled us to perform single and multiple QTL mapping strategies, leading to the detection of 65 QTLs with an influence on the formation of volatile metabolites, the production of extracellular main metabolites and general fermentation parameters. Our results confirm that multiple QTL mapping offers the possibility to detect additional, particularly minor loci. We were furthermore able to detect interacting QTLs for three evaluated traits, i.e., the formation of ethyl lactate, ethyl octanoate and propanol. However, it could be seen that an even larger number of segregants is required for a thorough and significant determination of QTL interactions.

We validated 13 genes in 9 QTLs, and of these genes, five (*AGP1*, *ALP1*, *FAS1*, *ILV6* and *LEU9*) have well described roles in metabolic pathways leading to yeast fermentative aroma formation. We could confirm their contribution to volatile production and characterized allelic variants that explain variations in these traits between the parent strains. Furthermore, the previously described fructophilic character of the MTF2621 allele of *HXT3* was confirmed in this study. For the other 7 validated genes (*AGP2*, *IXR1*, *MAE1*, *NRG1*, *RGS2*, *RGT1* and *SIR2*), we revealed contributions to the formation of fermentative aromas that were not previously reported. The fact that 5 of the 12 validated genes involved in volatile formation have broad regulatory functions on gene expression reveals the significant role of gene regulation in fermentative aroma production. These results demonstrate that QTL mapping is an effective and advisable approach for detecting the impact of globally acting genes on individual traits.

In summary, our findings of QTLs, their interactions and underlying gene variants emphasize the complexity of yeast fermentative aroma formation and provide the most extensive analysis of the links between genetic variation and the fermentative production of sensorial important volatiles to date. The results of this study will lead to the improvement of commercial *S. cerevisiae* starter cultures for the production of fermented food and beverages by non-GMO methods, such as marker-assisted breeding. As many of the described secondary metabolites are additionally used as biofuel additives or building blocks for chemical syntheses, improved knowledge about allelic variation may also open paths for improving strains in a wide range of biotechnological applications.

## Methods

### Media

Yeast was cultured at 28 °C in yeast extract peptone dextrose (YPD) media containing 10 g/L yeast extract, 20 g/L peptone and 20 g/L glucose. Solid YPD media contained 1.5% agar. Selective YPD media containing 200 μg/mL geneticin (G418), 200 μg/mL nourseothricin (clonNAT) or 200 μg/mL hygromycin B were used.

Wine fermentations were carried out in synthetic must (SM) described by Bely et al. (1990) [61]. The medium contains glucose and fructose (each 100 g/L) and assimilable nitrogen (200 mg/L) in the form of ammonium and free amino acids, which mimics the nitrogen content of standard grape juice.

### Yeast strains

The *S. cerevisiae* strains 4CAR1 and T73 exhibit different needs for nitrogen during wine fermentation, which may indicate different formations of aromas associated with nitrogen metabolism. The requirement was previously estimated using an approach based on the addition of nitrogen to keep the CO_2_ production rate constant during limitation of this substrate. In comparison to strain 4CAR1, strain T73 showed a higher need for nitrogen [22; strain T73 is coded as MTF1782 in this study]. The strain T73 belongs to the phylogenetic clade of wine strains, whereas strain 4CAR1 belongs to the group of champagne strains (Additional file 5), which originated through crossings between strains of the wine clade and the flor clade [28]. For the presented study, haploid spores of these strains were used, coded here as MTF2621 (haploid spore of strain 4CAR1 [Δ*HO*::*Neo*^r^]) and MTF2622 (haploid spore of strain T73 [Δ*HO*::*Nat*^r^]). The same spores have been previously used by Brice et al. (2014) to map QTLs influencing nitrogen requirement during fermentation [16, coded as MTF1782-B1 and MTF2029-C5 in this study].

### Generation of F2-segregants

The strains MTF2621 and MTF2622 were mated to form a zygote, which was selected on YPD-agar containing G418 and clonNAT. The zygote was then sporulated in liquid sporulation media using the protocol of Codon et al. (1995) [62]. The resulting tetrads were dissected into single spores to obtain the F1-generation using a Singer MSM 400 workstation (Singer Instruments). In most cases, only one spore per tetrad was taken for further experiments to increase genomic independence among the spores. The antibiotic resistance of the obtained spores was determined by growth assay on YPD-agar plates containing G418 or clonNAT. Two spores with different antibiotic properties were mated, and the formed zygotes were subsequently selected on YPD-agar containing G418 and clonNAT. These zygotes were sporulated and dissected again. In total, 130 single spores from the F2-generation were used for this study.

### Genotyping of strains

The genomic DNA of all 130 F2-segregants and both parent strains was isolated using the MasterPure™ Yeast DNA Purification Kit (epicentre) according to the protocol. The purity of the DNA was measured using a NanoDrop™ device (Thermo Fisher Scientific), and the concentration was determined by Qubit™ fluorometric quantification (Thermo Fisher Scientific). The DNA samples were then used for sequencing using Illumina technology (HiSeq 2500, paired end, 2 × 100 bp, sequencing platform Genotoul in Toulouse, France) at a sequencing depth of 20- to 80-fold. For each library, low-quality reads were processed and filtered with the FASTX Toolkit v0.0.13.2 and TRIMMOMATIC v0.30 [63] using a quality threshold of 20. First, reads were aligned to the S288C reference genome (release R64–1-1) using BWA v0.6.2 [64]. Once the reads were mapped, consensus genotype calling was performed using the tools available in the SAMtools package [65]. The global set of variants obtained in VCF format contained 18,155 biallelic variant positions with a genotyping quality greater than 100. The effect of SNPs on putative transcription factor binding sites was analyzed using YEASTRACT (release 2017; [66]). For the location of SNPs in annotated protein domains, information available in the *Saccharomyces* Genome Database (http://www.yeastgenome.org) was used. The initial variant set was filtered to ensure a minimum spacing of 2.0 kb between SNPs. This resulted in a genotyping variant dataset of 3727 SNP markers (Additional file 6). To increase the meaningfulness of the analysis, four strains with the most ambiguous markers were excluded. One strain was excluded because it was too close in genomic proximity to another segregant. This left a population of 125 F2-segregants for statistical analyses.

### Phenotyping of strains

Segregants were fermented in duplicate with the parent strains as controls. The strains were grown overnight in 50 mL of YPD media. The cell density was determined using a Multisizer™ 3 Coulter Counter (Beckman Coulter). Sterilized 300-mL glassware mini fermenters were filled with 280 mL of SM200 and closed with an air lock. The fermenters were inoculated to a cell density of 1 × 10^6^ cells/mL, weighed and left at 24 °C under stirring (300 rpm).

To determine the concentration of aroma compounds, a sample was taken when approx. 80% of the sugars were depleted. This corresponded to 67.9–75 g/L produced CO_2_ and was determined by weighting the fermenters regularly to draw the weight decrease caused by the release of CO_2_. Volatiles were extracted with dichloromethane according to the method described by Rollero et al. (2015) [67]. The concentrations of fermentative aromas were measured via GC/MS on full scan mode using a DB-WAX 60m GC column. Thirty-four compounds were quantified using internal deuterated standards. In addition, the concentrations of extracellular metabolites after 80% of the fermentation were measured using HPLC (REZEX™ ROA-Organic Acid H+ (8%), 0.005 M H_2_SO_4_).

### QTL mapping

The data obtained from phenotyping and genotyping were used to identify QTLs in the genome of yeast strains that influence the formation of volatile secondary metabolites during wine fermentation. Furthermore, QTLs influencing fermentation parameters, substrate consumption and the production of extracellular main metabolites were examined. The statistical analyses were performed using the programming language R v3.2.3 (www.r-project.org) with the R/qtl v1.40–8 and R/eqtl v1.1–7 libraries [68]. QTL mapping was performed with two different phenotype models, the normal model using Haley-Knott regression and a non-parametric analysis, resulting in logarithm of odds (LOD) scores for each marker and pseudo-marker every 2.5 cM (interval mapping method). An interval estimate of the location of each QTL was obtained as the 1-LOD support interval, the region in which the LOD score is within 1 unit of the peak LOD score. If the same locus was detected with both models, the results with the higher LOD score were selected. A two-dimensional, two-QTL scan was performed using the function *scantwo*. Multiple QTL mapping was performed twice with the function *stepwiseqtl*, once with strictly additive models and once with models that allowed for interactions. The limit of detected QTLs was set to 5. Newly detected QTL positions were counted when the LOD scores of models including these loci were higher than the added LOD score penalties of combining all loci of the respective model. For each method used, individual LOD score thresholds for a false discovery rate of 0.05 were determined with 1000 permutations. QTL mapping results for single traits were grouped as common QTL regions if their peaks were less than 10 cM apart. Proposed models of interaction were further assessed with the function *fitqtl*. The support of individual terms was evaluated by dropping each QTL from the proposed model, one at a time, and comparing the resulting models to the full model.

### Reciprocal hemizygosity analysis

Validation of found QTLs was performed using reciprocal hemizygosity analysis (RHA) [12]. QTLs were either evaluated as a whole region or single genes with a potential influence on the trait were tested. For the deletion of selected regions, the parent strains were mated to form the heterozygote. Subsequently, one allele of the region was deleted randomly by homologous recombination with a disruption cassette containing the hygromycin B resistance gene (*hph*^r^) that was obtained by PCR of the plasmid pAG32 (addgene) with the primers del_(QTL)_fw and del_(QTL)_rv (Additional file 7). Positive integration was selected by plating the transformed cells on YPD-agar plates containing hygromycin B. Correct deletion of the region was verified by PCR using primer test_(QTL)_fw that binds upstream of the deleted region and primer Hygro_rv that binds within the deletion cassette. The remaining allele of the QTL was identified by allelic PCR using primer test_(QTL)_fw that binds upstream of a selected gene in the hemizygous region and primers tal_(QTL)_1 or tal_(QTL)_2 that bind at a SNP position within the same gene.

For the deletion of single genes, the sequences were deleted in both parent strains by homologous recombination with a disruption cassette containing the hygromycin B resistance gene (*hph*^r^) that was obtained by PCR of the plasmid pAG32 with the primers del_(GENE)_fw and del_(GENE)_rv. Positive integration was selected by plating the transformed cells on YPD-agar plates containing hygromycin B. Correct deletion of the gene was verified by PCR using primer test_(GENE)_fw that binds upstream of the deleted gene and primer Hygro_rv that binds within the deletion cassette. Deleted parent strains were subsequently mated with the opposite undeleted parent to form a heterozygote that is hemizygous for the target gene.

Hemizygous constructions were phenotyped in triplicate. The significance of the influence of an allelic target region or gene variant on the trait was evaluated by student’s t-test. If the impact of a variant on several traits was tested, *p*-values were not adjusted for multiple comparisons.

## Additional files

Additional file 1: Additional phenotypic information. Concentrations of determined secondary metabolites produced by the parental strains used in this study with trait variety among the segregant population given as interquartile range (IQR) and heritability of evaluated traits. (XLSX 21 kb)

Additional file 2: Phenotype distributions among population. Distribution of evaluated traits for QTL mapping among all 130 F2-segregants of the study. The position of parental cells within the population is marked in red for MTF2621 and in green for MTF2622. (PDF 12 kb)

Additional file 3: Additionally detected allelic effects of the described enzymes as determined by RHA. Allelic effect of the sugar transporters Hxt3, Hxt6 and Hxt7 on the G/F ratio (**A**). Allelic effect of the enzymes Mae1 and Sir2 on the acetate yield (**B**). Allelic effect of the enzymes Agp1 and Mae1 on the production of 2-phenylethanol (**C**). Allelic effect of Mae1 on the formation of ethyl lactate (**D**) and fatty acids and fatty acid ethyl esters (**E**). Concentrations are given in relation to the heterozygote of the parental strains MTF2621 and MTF2622. (*p*-value: ns (not significant) > 0.05, * ≤ 0.05, ** ≤ 0.01, *** ≤ 0.001). (PDF 9 kb)

Additional file 4: SNPs in predicted regulatory binding sites of validated genes. Detected SNPs in the 1000-bp upstream region of evaluated target genes that affect binding motifs for regulatory proteins as predicted with YEASTRACT [66]. Comparison of the strains MTF2621 and MTF2622 with the *S. cerevisiae* reference strain S288C. (XLSX 242 kb)

Additional file 5: Genomic background of parent strains. Location of the *S. cerevisiae* strains used in this study, MTF2621 (4CAR1) and MTF2622 (T73), within the genotypic subgroups of champagne strains (light green lines) and wine strains (dark green lines). Phylogenetic tree constructed with data from and as described by Legras et al. (2007) [69]. (PDF 2648 kb)

Additional file 6: Marker map. Graphic representation of marker positions that were used for linkage analysis. (TIF 8 kb)

Additional file 7: Table of primers used in this study. (XLSX 295 kb)

## Abbreviations

FAS: Fatty acid synthetase
LOD: logarithm of odds
PCA: Principal component analysis
QTL: Quantitative trait locus
RHA: Reciprocal hemizygosity analysis
SM: Synthetic must
SNP: Single-nucleotide polymorphism
YPD: Yeast extract peptone dextrose
