# Supplementary figures and images for: QTL mapping of volatile compound production in Saccharomyces cerevisiae during alcoholic fermentation
Source: BMC Genomics. 2018 Mar 1;19:166. doi: 10.1186/s12864-018-4562-8 (PMC5831830; doi:10.1186/s12864-018-4562-8)

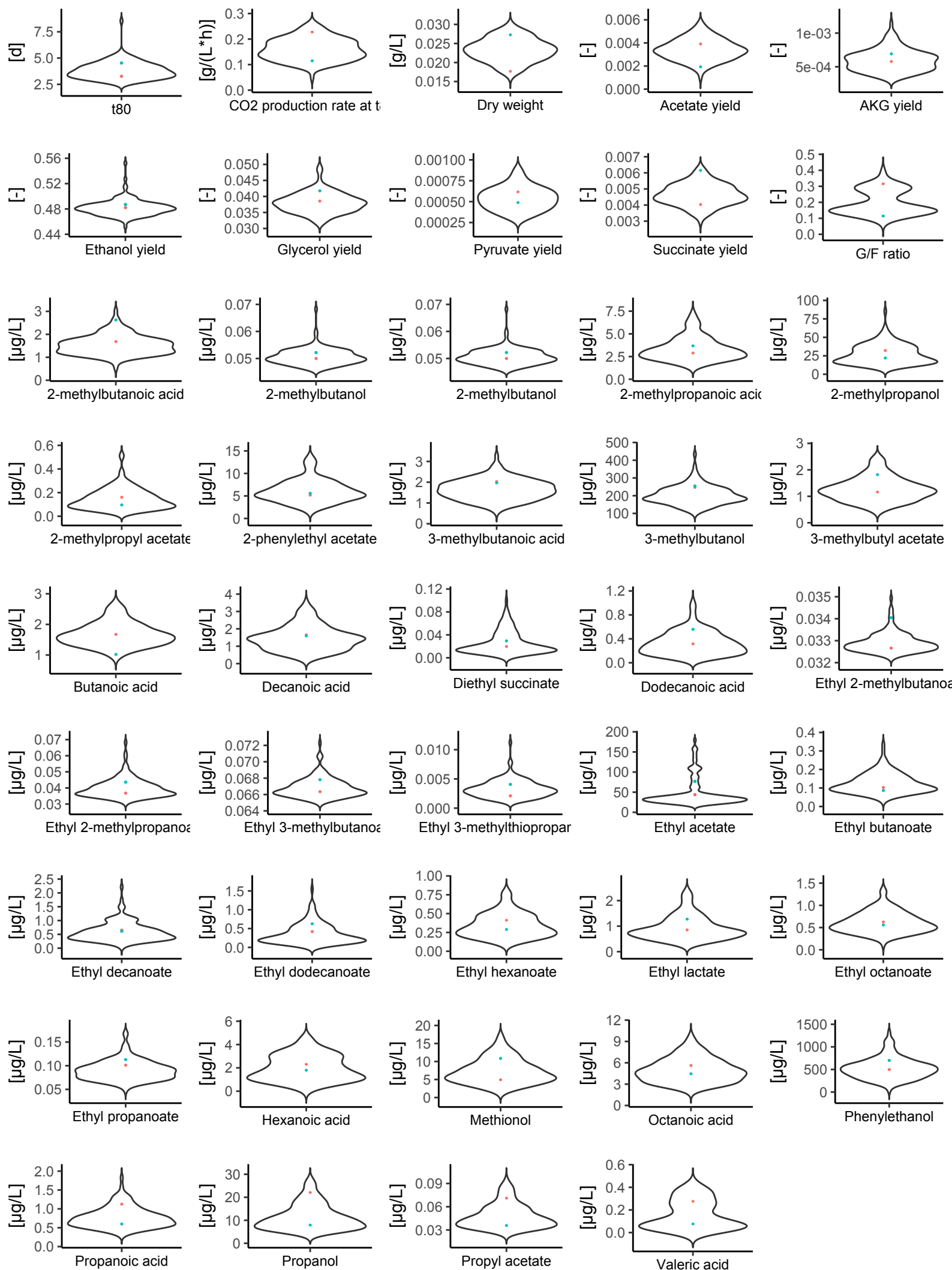

Strain ● MTF2621 ● MTF2622

Supplement: Supplementary file 2 — Phenotype distributions among population. Distribution of evaluated traits for QTL mapping among all 130 F2-segregants of the study. The position of parental cells within the population is marked in red for MTF2621 and in green for MTF2622. (PDF 12 kb) [file 12864_2018_4562_MOESM2_ESM.pdf]

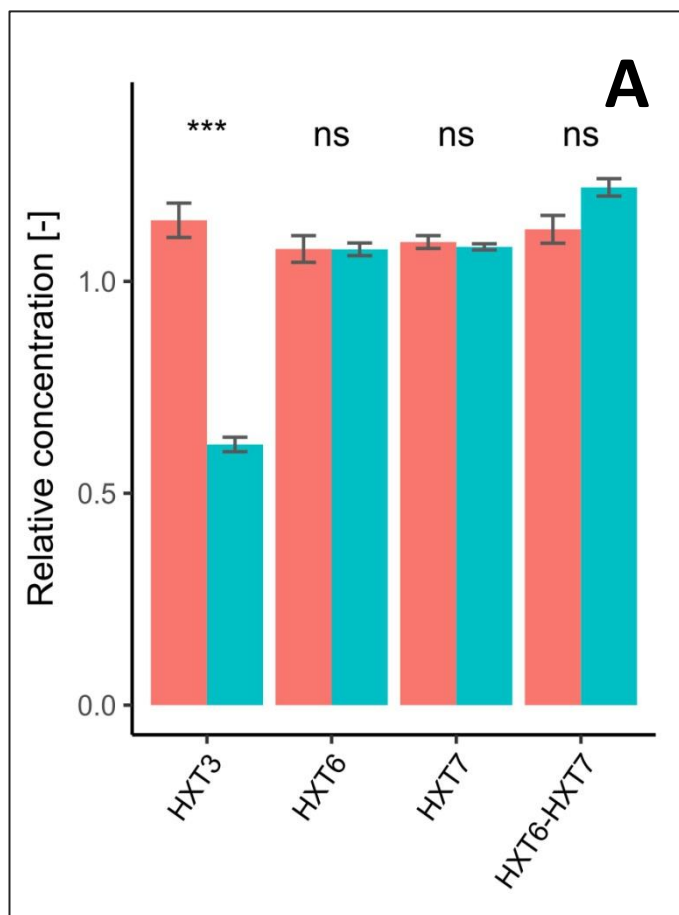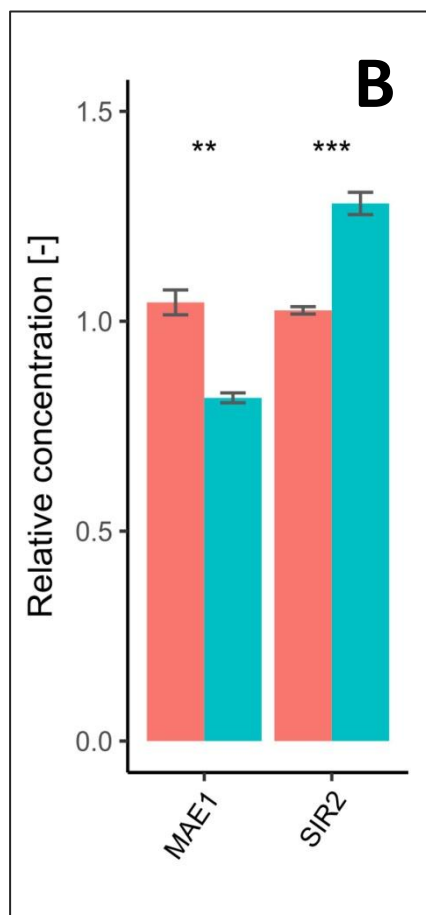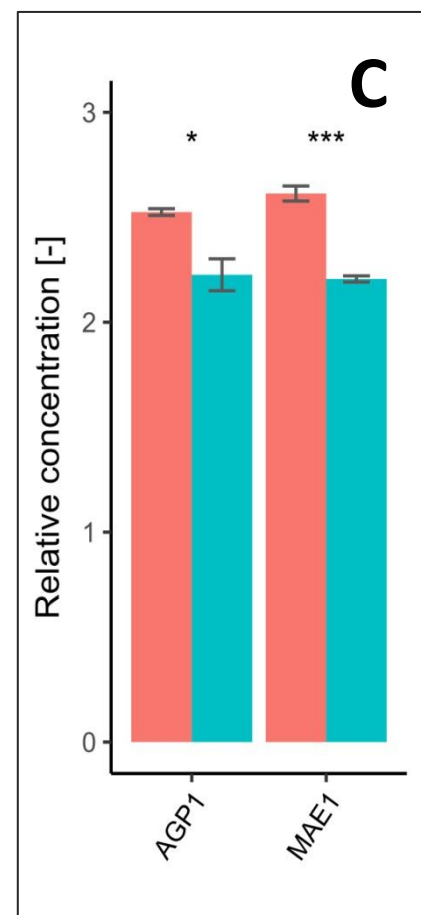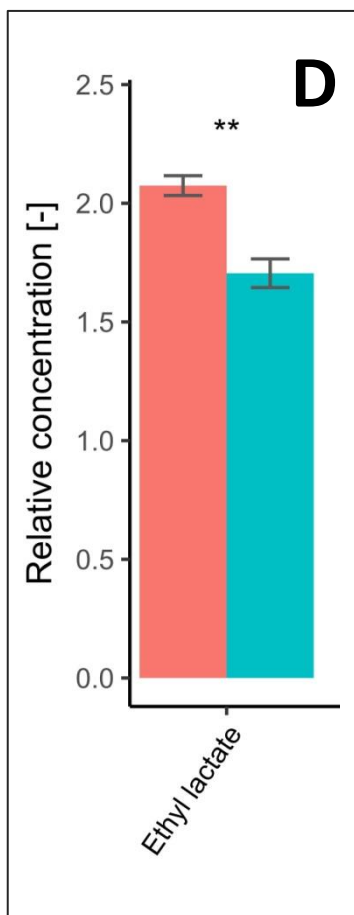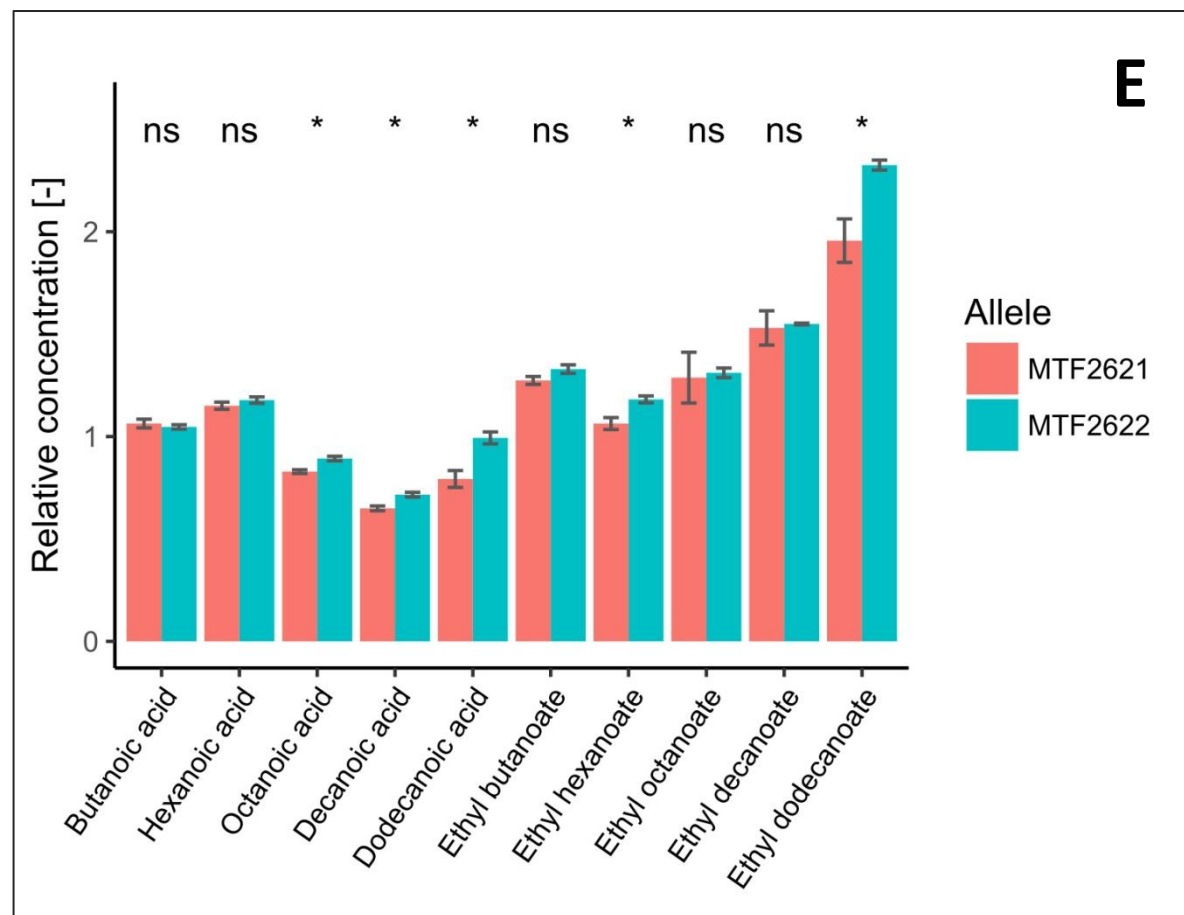

Supplement: Supplementary file 3 — Additionally detected allelic effects of the described enzymes as determined by RHA. Allelic effect of the sugar transporters Hxt3, Hxt6 and Hxt7 on the G/F ratio (A). Allelic effect of the enzymes Mae1 and Sir2 on the acetate yield (B). Allelic effect of the enzymes Agp1 and Mae1 on the production of 2-phenylethanol (C). Allelic effect of Mae1 on the formation of ethyl lactate (D) and fatty acids and fatty acid ethyl esters (E). Concentrations are given in relation to the heterozygote of the parental strains MTF2621 and MTF2622. (p-value: ns (not significant) > 0.05, * ≤ 0.05, ** ≤ 0.01, *** ≤ 0.001). (PDF 9 kb) [file 12864_2018_4562_MOESM3_ESM.pdf]

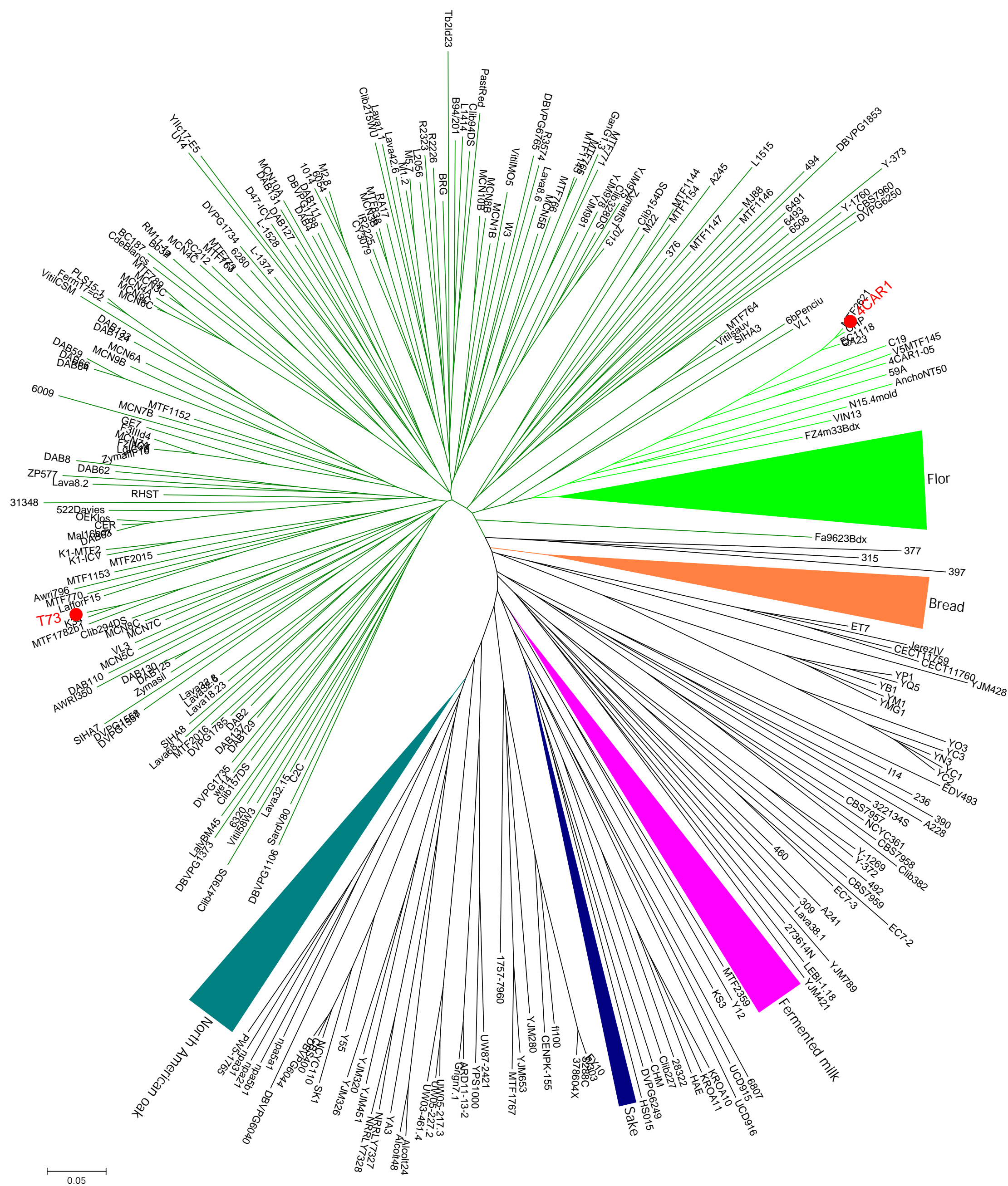

0.05

Supplement: Supplementary file 5 — Genomic background of parent strains. Location of the S. cerevisiae strains used in this study, MTF2621 (4CAR1) and MTF2622 (T73), within the genotypic subgroups of champagne strains (light green lines) and wine strains (dark green lines). Phylogenetic tree constructed with data from and as described by Legras et al. (2007) [69]. (PDF 2648 kb) [file 12864_2018_4562_MOESM5_ESM.pdf]

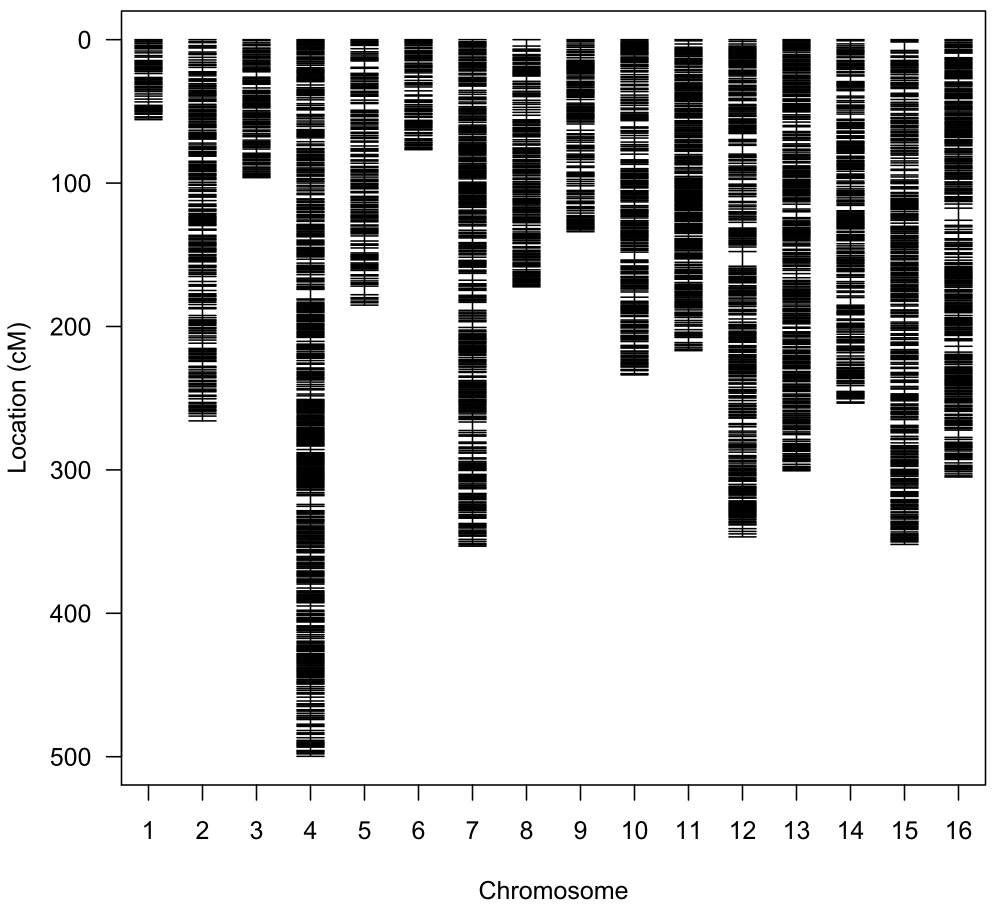

Supplement: Supplementary file 6 — Marker map. Graphic representation of marker positions that were used for linkage analysis. (TIF 8 kb) [file 12864_2018_4562_MOESM6_ESM.tif]
